# Angiotensin Converting Enzyme Inhibitors and Angiotensin Receptor Blockers Rescue Memory Defects in *Drosophila*-Expressing Alzheimer’s Disease-Related Transgenes Independently of the Canonical Renin Angiotensin System

**DOI:** 10.1523/ENEURO.0235-20.2020

**Published:** 2020-12-17

**Authors:** Shin-Hann Lee, Sarah M. Gomes, Judy Ghalayini, Konstantin G. Iliadi, Gabrielle L. Boulianne

**Affiliations:** 1 Program in Developmental and Stem Cell Biology, The Hospital for Sick Children, M5G 0A4, Toronto, ON, CA; 2 Department of Molecular Genetics, University of Toronto, M5S 1A1, Toronto, ON, CA; 3 Institute of Medical Science, University of Toronto, M5S 1A1, Toronto, ON, CA

**Keywords:** Alzheimer’s disease, amyloid, *Drosophila*, memory, presenilin, renin angiotensin system

## Abstract

Alzheimer’s disease (AD) is a degenerative disorder that causes progressive memory and cognitive decline. Recently, studies have reported that inhibitors of the mammalian renin angiotensin system (RAS) result in a significant reduction in the incidence and progression of AD by unknown mechanisms. Here, we used a genetic and pharmacological approach to evaluate the beneficial effects of angiotensin converting enzyme inhibitors (ACE-Is) and angiotensin receptor blockers (ARBs) in *Drosophila* expressing AD-related transgenes. Importantly, while ACE orthologs have been identified in *Drosophila*, other RAS components are not conserved. We show that captopril, an ACE-I, and losartan, an ARB, can suppress a rough eye phenotype and brain cell death in flies expressing a mutant human *C99* transgene. Captopril also significantly rescues memory defects in these flies. Similarly, both drugs reduce cell death in *Drosophila* expressing human *A*β*42* and losartan significantly rescues memory deficits. However, neither drug affects production, accumulation or clearance of *A*β*42*. Importantly, neither drug rescued brain cell death in *Drosophila* expressing human Tau, suggesting that RAS inhibitors specifically target the amyloid pathway. Of note, we also observed reduced cell death and a complete rescue of memory deficits when we crossed a null mutation in *Drosophila Acer* into each transgenic line demonstrating that the target of captopril in *Drosophila* is Acer. Together, these studies demonstrate that captopril and losartan are able to modulate AD related phenotypes in the absence of the canonical RAS pathway and suggest that both drugs have additional targets that can be identified in *Drosophila*.

## Significance Statement

Alzheimer’s disease (AD) is a devastating neurodegenerative disorder for which there is no cure. Recently, studies have reported a significant reduction in the incidence of AD and dementia among patients taking angiotensin converting enzyme inhibitors (ACE-Is) and angiotensin receptor blockers (ARBs). Given the enormous and immediate potential of ACE-Is and ARBs for AD therapeutics, it is imperative that we understand how they function and why they are beneficial in some patients but not others. Here, we show that captopril, an ACE-I, and losartan, an ARB, can restore memory defects in flies expressing human AD transgenes in the absence of the canonical renin angiotensin system (RAS) pathway. These studies provide us with a unique opportunity to identify novel targets of ACE-Is and ARBs and evaluate their therapeutic effectiveness in robust models of AD.

## Introduction

Alzheimer’s disease (AD) is a degenerative disorder of the central nervous system that causes progressive memory and cognitive decline during mid to late adult life. Mutations in three genes, *APP*, *presenilin 1*, and *presenilin 2* (*PS1* and *PS2*), cause early-onset autosomal dominant AD, which accounts for <5% of AD cases (Goate et al., 1991). APP encodes a single-pass transmembrane protein that is cleaved by two proteases, β-secretase and γ-secretase, to generate amyloid peptides. PSs encode the catalytic component of γ-secretase (Wolfe et al., 1999), which cleaves the C-terminal fragment of APP (APP-CTF, C99) to produce Aβ peptides. Generally, longer Aβ peptides (Aβ42) are prone to self-aggregation and are concentrated in amyloid plaques, which are associated with brain atrophy, regional hypometabolism, network dysfunction, inflammation, and oxidative stress (Holtzman et al., 2011). Therefore, Aβ42 and plaques are often used as a diagnostic tool for AD prognosis and progression (Hansson et al., 2007; Lewczuk et al., 2015).

Recently, biochemical studies have shown that additional proteins can associate with PS and γ-secretase to modulate its assembly and/or interaction with specific targets (Bursavich et al., 2016; Tan et al., 2016). Proteins that modulate γ-secretase assembly would provide valuable insight into the function of this important complex during development and disease. Similarly, proteins that modulate the interaction of γ-secretase with specific targets such as APP, or affect the production of Aβ peptides or their clearance, might allow for the development of new therapeutic targets for AD. Although extremely promising, only a few PS and γ-secretase modulators have been identified and their mechanism of action remains largely unknown.

Using a genetic approach in *Drosophila*, we previously identified *Acer* and *Ance-5,* two orthologs of human angiotensin converting enzyme (ACE), as modifiers of PS and C99 (van de Hoef et al., 2009). ACE is a metalloprotease that cleaves angiotensin 1, a major component of the renin angiotensin system (RAS) that regulates blood pressure in humans. Importantly, while ACE orthologs have been identified in *Drosophila*, other components of the RAS are not conserved. Interestingly, several studies have established a link between RAS-targeting anti-hypertensive drugs, such as angiotensin converting enzyme inhibitors (ACE-Is) and angiotensin receptor blockers (ARBs), and AD (Ohrui et al., 2004; Davies et al., 2011; Abdalla et al., 2013; Qiu et al., 2013; Yasar et al., 2013; de Oliveira et al., 2014; Wharton et al., 2015). For example, both ACE-Is and ARBs have been shown to delay the onset of cognitive impairment and neurodegeneration in mouse models of AD and in some patients, although the mechanism of action remains unclear (Alvarez et al., 1999; Ohrui et al., 2004; Hajjar et al., 2005; Edwards et al., 2009; Miners et al., 2009; Belbin et al., 2011; Qiu et al., 2013; Soto et al., 2013; Yasar et al., 2013; de Oliveira et al., 2014; Kauwe et al., 2014; O’Caoimh et al., 2014; Wharton et al., 2015; Ho et al., 2017).

Here, we have examined the effects of ACE-Is and ARBs in *Drosophila* that express human AD-related transgenes. We show that captopril, an ACE-I and losartan, an ARB, suppress a rough eye phenotype and cell death in the brains of flies expressing a human C99 transgene carrying a London mutation. Moreover, captopril significantly rescues memory deficits in these flies. Similarly, both drugs reduce cell death and losartan significantly rescues memory deficits in *Drosophila* expressing human Aβ42. Importantly, neither drug affects the levels or clearance of Aβ42. We also observed no effects of either drug on degenerative phenotypes observed in *Drosophila* expressing human Tau, suggesting that the beneficial effects are specific to APP-CTF and Aβ42 expressing flies. Importantly, we found that an *Acer* null mutant was able to rescue cell death and memory deficits in *Drosophila* expressing Aβ42 consistent with Acer being the target of captopril in *Drosophila*. However, since the downstream targets of Acer including angiotensin and the angiotensin receptor are not conserved, we could not use a similar approach to identify the target/s of losartan. Together, these studies demonstrate that captopril and losartan are able to modulate AD related phenotypes in *Drosophila*. Moreover, since these beneficial effects are observed in the absence of the canonical RAS, these studies suggest that captopril and losartan may have additional targets that can be identified in *Drosophila*.

## Materials and Methods

### *Drosophila* stocks

Stocks and crosses were maintained on standard media with or without drug treatment at 29°C for eye models and at 25°C for CNS models with 65% relative humidity and a 12/12 h light/dark cycle. *gmr-GAL4;UAS-mCD8GFP/SM5CyO* recombinant line was generated as described (Burr et al., 2014; referred to as *gmr-GAL4-UAS-GFP*). *UAS-APP^C99J4^*, *UAS-APP^C99J6^* (referred to as UAS-*C99^wt^*), and *UAS-APP^C99V717I^*London mutation (referred to as UAS-*C99^V717I^*) have been previously described (Finelli et al., 2004). *elav-GAL4/CyO* (8765), *elav-GAL4^C155^*(458), *UAS-APP^Abeta42.B^* (33769; referred to as *UAS-Aβ42*), *UAS-Tau^wt1.13^* (51362; expresses the 2N4R isoform of human Tau referred to as *UAS-Tau*), *w^1118^* and *Canton-S* (referred to as wt) were obtained from the Bloomington Stock Center. The *Acer* null allele (*Acer^Δ168^*) was obtained from (Carhan et al., 2011) and crossed to *elav-GAL4^C155^, UAS-APP^C99V717I^* and *UAS-Aβ42* flies to generate fly lines expressing AD-related transgenes with an *Acer* null mutation. *elav-GAL4^C155^*driver was used instead of *elav-GAL4/CyO* for *Acer* null-related experiments for the purpose of generating a homozygous *Acer* null mutation.

### Drug treatments

All adult flies were maintained on standard media with or without addition of either captopril (5 mm; Sigma-Aldrich) or losartan (1 mm; US Pharmacopeial Convention) from the first day after eclosion (DAE = 0).

### GFP and REP imaging

Heads from 7-d-old adults were removed using spring scissors and slide mounted using double-sided tape. Heads were imaged at room temperature using a confocal Leica TCS SP5 microscope (Leica Microsystems Inc.), with 20× objective and standard GFP filters with Leica Application Suite (LAS X) software (Leica Microsystems Inc.). Images were processed using ImageJ (Rasband, W.S., ImageJ, NIH; http://imagej.nih.gov/ij/, 1997–2016). GFP expression was analyzed using corrected total cell fluorescence (CTCF) calculations (based on Burgess et al., 2010). Rough eye phenotype images were captured with a 4× objective using a Nixon SMZ-2T light microscope and an OptixCam Summit K2 microscope camera with ToupView software (by ToupTek Photonics).

### Terminal deoxynucleotidyl transferase-mediated biotinylated UTP nick end labeling (TUNEL) labeling

Brains from 28-d-old adults were dissected in cold PBS with 0.5% Triton X-100 and fixed in 4% paraformaldehyde at room temperature for 30 min. Brains were then rinsed twice in PBS with 0.5% Triton X-100 for 10 min each and washed once in H_2_O plus 0.5% Triton X-100 and 0.1% sodium citrate solution for 15 min at 4°C followed by two washes in PBS with 0.5% Triton X-100 for 10 min each. TUNEL staining was performed according to the manufacturer instructions (Roche, *in situ* cell death detection kit, catalog #11684795910). Images were captured as a Z-stack and compressed into a single image using a Nikon A1R confocal microscope. Cell death was manually counted for statistical analysis.

### Courtship conditioning assay

All experiments and analyses were performed double-blind as previously described (Kamyshev et al., 1999). Experimental flies were collected within 6 h after eclosion and kept individually in culture vials on standard media with or without drugs (captopril or losartan) for 28 d until the experiment was performed. One day before the experiment, *Canton-S* virgin females were mated with same age males. Mated females were then used for training and testing. All behavioral experiments were performed within a 3-h time window (between 4 and 7 P.M.) in an environmental control room. Male courtship behavior was observed in a custom-made Perspex chamber (15-mm diameters, 5 mm high) with a sliding opaque partition that divided the chamber into two halves, with two lateral entries (3-mm diameter) with stoppers. Before training or testing, each chamber was cleaned with 50% ethanol and dried. For training, a naive male (with no sexual experience) was placed into an experimental chamber together with a 5-d-old mated *Canton S* female. After several minutes to recover from the transfer the divider was withdrawn and the flies were left together for 1 h. After training, an experimental male was isolated for 30 min and then tested for short-term memory (STM) performance with a mated female during 10 min. Courtship behavior during the test session was video recorded using a color camera (EverFocus EQ.610, Polistar II) that was fitted with a CCTV lens (Computar, VariFocal TG4Z2813 FCS-IR) and fixed on a mounting bracket ∼50 cm above the chamber. The distance of the camera to the object as well as the zoom, focus and iris aperture were optimized for video recording. Subsequent video analysis of time spent performing courtship behavior and all statistical comparisons were done using computer software (*Drosophila* Courtship Lite 1.4, developed by N. G. Kamyshev, Russian Academy of Science). Courtship index (CI) was defined as the percentage of time spent performing courtship behavior during the observation period. Memory index (MI) was calculated as: [100 [1 – (CI with training/mean of CI without training)] (Kamyshev et al., 1999; Lim et al., 2018).

### Western blottings

Ten heads (five male, five female) from 7- and 28-d-old adults were lysed in 2× tricine sample buffer (Bio-Rad catalog #1610739), boiled for 5 min, and run on 16.5% Tris-tricine gels (Bio-Rad catalog #4563066) with 1× SDS/Tris/tricine running buffer (Bio-Rad catalog #1610744). Protein was transferred onto 0.2-μm nitrocellulose membranes (Bio-Rad catalog #1620168) using standard transfer buffer. Membranes were boiled 3 min in 1× PBS then blocked for 1 h using 1× TBST with 5% skim milk. Primary antibody detection was done overnight at 4°C using Aβ-6E10 (1:500; Biolegend catalog #803001) and anti-α-tubulin (1:1000) or anti-β-actin (1:1000) in 1× TBST 5% skim milk. Membranes were washed 3× in 1× TBST for 10 min each. Secondary antibody detection was done using anti-mouse-horseradish peroxidase for 2 h at 4°C (1:10,000). Membranes were then washed 3× in 1× TBST for 10 min each. Signal was detected using chemiluminescence substrates (Bio-Rad catalog #1705060) and membranes were imaged using LI-COR Odyssey Fc imager.

### ELISA assays

Aβ42 peptide levels were determined using human Aβ specific ELISA kits (Invitrogen, catalog #3441) as per manufacturer’s instructions. Forty heads from 28-d-old maintained at 25°C were lysed in 1× RIPA buffer with a complete protease inhibitor (Roche) containing 50 mm Tris, 150 mm NaCl, 1% SDS, 1% NP-40, and 0.5% sodium deoxycholate, pH 8.0. The homogenates were diluted twofold before loading onto the plate. The signals were measured at 450 nm using a microplate reader. The whole experiment was performed as described previously (Van de Hoef et al., 2009).

### Plaque staining

Flies expressing *Aβ42* in the CNS were maintained on standard media with or without drugs (captopril or losartan) for 28 d after eclosion and subjected to plaque staining using the amyloid specific luminescent conjugated oligiohiophene (LCO), p-FTAA, as previously described (Jonson et al., 2018). Fly brains were dissected in cold PBS and fixed in 96% ethanol for 10 min. Samples were then rehydrated following a step wash with 70%, 50%, 0% ethanol, then washed with PBS and stained with p-FTAA diluted 1:1000 in PBS for 30 min. After incubation with p-FTAA, samples were washed in PBS and mounted using DAKO mounting medium. Z-stack images of whole brains were acquired using a Sp8 confocal microscope and images were analyzed using Volocity Software. Levels of amyloid deposits were determined by measuring total pixel count over set threshold across z-stacks.

### Statistics

Statistical analyses were done using GraphPad Prism or SPSS. Two-tailed Student’s *t* test was used to analyze differences between two groups. One-way ANOVA with Bonferroni *post hoc* analysis was used for multiple comparisons. Kruskal–Wallis ANOVA followed by Dunn’s multiple comparisons *post hoc* test were used for non-parametric analyses. Data are graphically reported as mean ± SEM. Kruskal–Wallis ANOVA test followed by Dunn’s multiple comparisons test and Mann–Whitney *U* test were used for statistical comparisons for the courtship conditioning assay. Data are graphically reported as mean/median, and the box-and-whisker plots for CIs show 10th, 25th, 75th, and 90th percentiles. MIs are shown as mean ± SEM.

## Results

### Characterization of *C99^wt^, C99^V717I^*, and *Aβ42* phenotypes

To determine whether pharmacological inhibition of the RAS pathway using ACE-Is and ARBs can exert any beneficial effects in fly models of AD, we used the *GAL4-UAS* system to target expression of human AD-related transgenes in the compound eye and CNS of *Drosophila* (Brand and Perrimon, 1993). Previous studies have shown that expression of these transgenes in the compound eye results in a rough eye phenotype, characterized by changes in the size of the eye that can be because of changes in photoreceptor neurons, loss of interomatidial bristles and pigmentation, and necrotic tissue (Prüßing et al., 2013; Iyer et al., 2016). Expression of AD-related transgenes in the CNS has also been shown to lead to Aβ aggregation, plaque formation, neurodegeneration, shortened lifespan, and deficits in learning and memory (Ye and Fortini, 1999; Finelli et al., 2004; Greeve et al., 2004; Iijima et al., 2004, 2008; Chakraborty et al., 2011; Prüßing et al., 2013).

**Figure 1.**
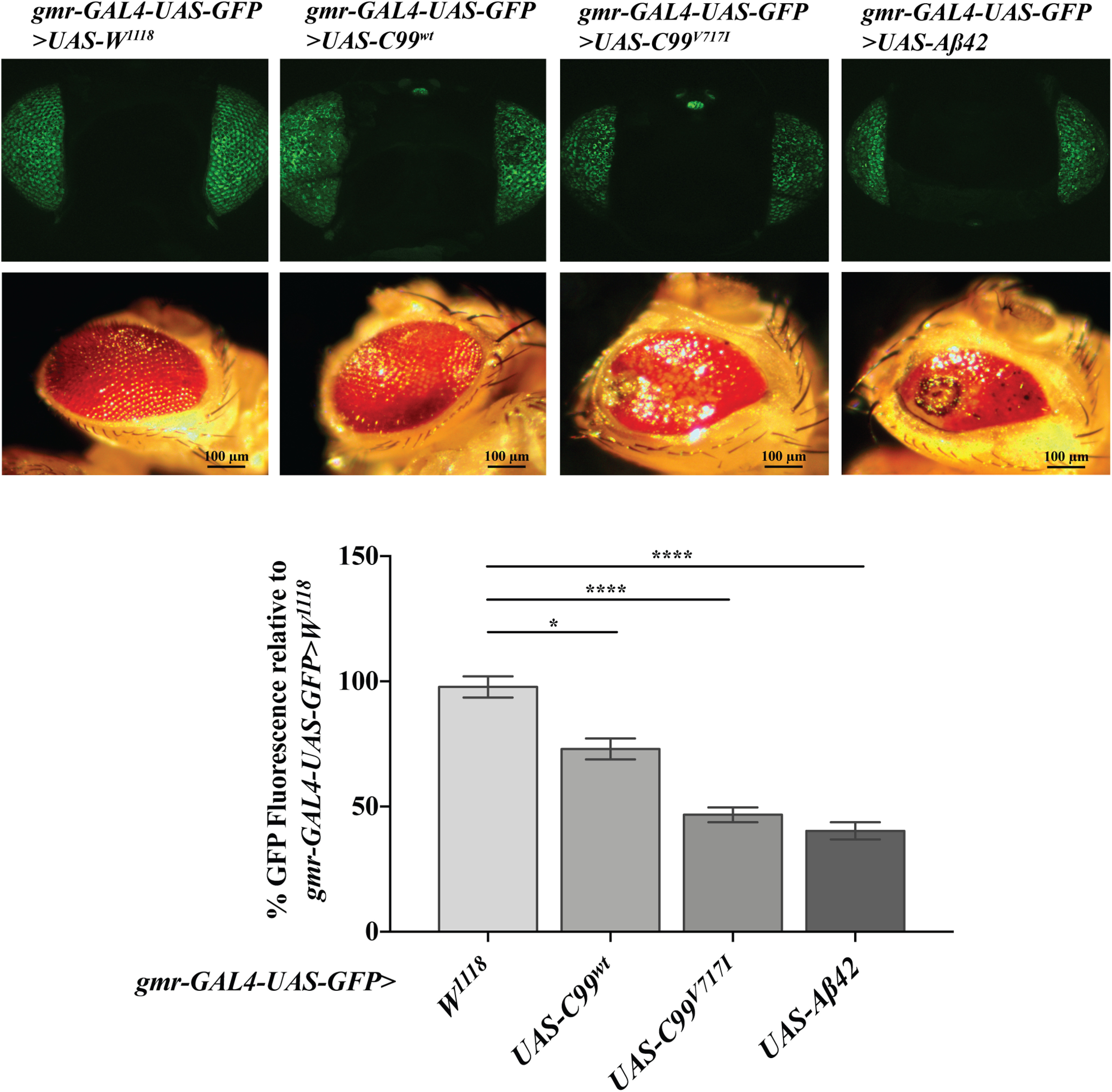
*gmr-GAL4 Drosophila* model of AD. Confocal GFP (top row) and light microscope (bottom row) images of 7-d-old *gmr-GAL4-UAS-GFP>w^1118^*, *gmr-GAL4-UAS-GFP>UAS-C99^wt^*, *gmr-GAL4-UAS-GFP>UAS-C99^V717I^*, and *gmr-GAL4-UAS-GFP>UAS-Aβ42* fly heads as labeled. Kruskal–Wallis ANOVA analysis of GFP quantification showed significant differences between transgenes (*p *<* *0.0001). Multiple comparison analysis using Dunn’s corrected multiple comparison test showed flies expressing *C99^wt^* (*N *=* *41), *C99^V717I^* (*N *=* *56), and *Aβ42* (*N *=* *30) have a significant decrease in GFP expression compared with *wt* (*N *=* *88; *p *=* *0.0388, *p *<* *0.0001, and *p *<* *0.0001, respectively). Data are shown as mean ± SEM; *p <* *0.05, ****p < 0.0001.

To quantitate the rough eye phenotype generated by expression of human AD-related transgenes, we crossed each UAS-transgenic line with flies expressing membrane bound UAS-GFP to a *gmr-GAL4* driver that targets expression in the developing eye. In previous studies, GFP intensity has been shown to be negatively correlated with retinal cell death (Burr et al., 2014). We found that expression of both *gmr>C99^V717I^* and *gmr>Aβ42* resulted in a significant decrease in mean GFP intensity (46.67 ± 2.96% and 40.32 ± 3.39%, respectively) compared with a driver-control (97.82 ± 4.22%; Fig. 1), while expression of *gmr>C99^wt^* showed intermediate levels of GFP intensity (73.01 ± 4.15%) compared with controls (Fig. 1).

**Figure 2.**
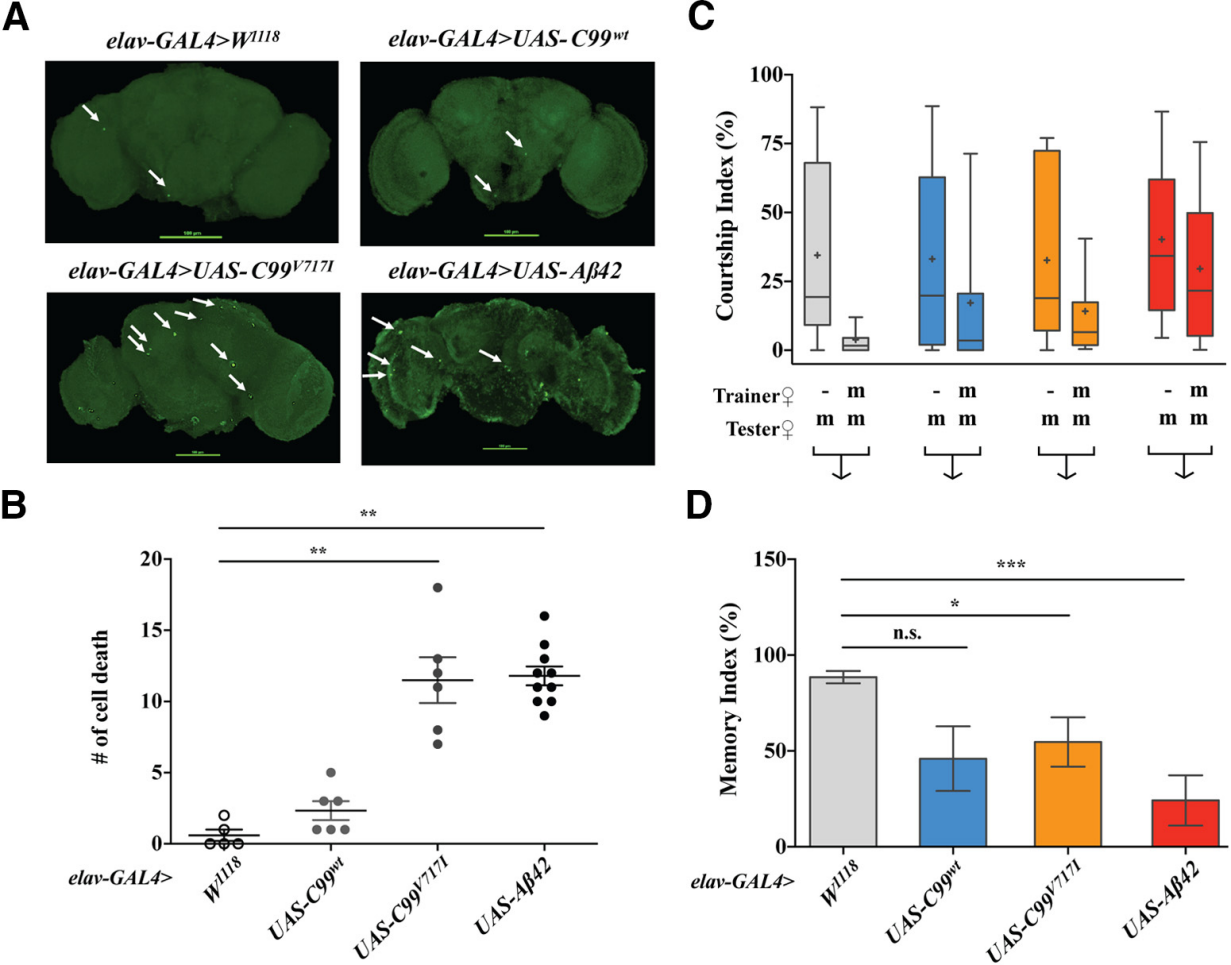
*elav-GAL4 Drosophila* model of AD. ***A***, TUNEL labeling in brains of 28-d-old flies. ***B***, Kruskal–Wallis ANOVA analysis with Dunn’s multiple comparisons test showed that flies expressing *C99^V717I^*(*N *=* *6) and *AB42* (*N *=* *10) have a significant higher amount of TUNEL-labeled cell death compared with *wt* (*N *=* *5; *p *=* *0.0091, *p *=* *0.0015, respectively). ***C***, CIs were calculated by dividing the time a male spent in courtship to a total given time. Trainer and tester females: –, none; m, mated female. Box-and-whisker plots for CI show 10th, 25th, 75th, and 90th percentiles and mean (+). ***D***, MIs were calculated as [100 [1 – (CI with training/mean of CI without training)]. Kruskal–Wallis ANOVA test followed by Dunn’s multiple comparisons test were used for statistical comparisons (*N ≥ *20 for each genotype). *elav-GAL4>UAS-C99^V717I^* and *elav-GAL4>UAS-Aβ42* flies showed statistically significant lower MIs when compared with *elav-GAL4>w^1118^* but not *elav-GAL4>UAS-C99^wt^* (*p *=* *0.0423, *p *=* *0.0001, and *p *=* *0.1859, respectively) Data are shown as mean ± SEM; *p <* *0.05, **p <* *0.01, ***p <* *0.001; n.s., not significant.

We also examined the pathologic effects associated with expression of human AD transgenes in the CNS using the pan-neuronal *elav*-*GAL4* driver (Fig. 2). We first examined brain cell death using TUNEL analysis and found that expression of *elav>C99^V717I^* or *elav>Aβ42* resulted in a significant increase in cell death within the adult brain (11.5 ± 1.6 and 11.8 ± 0.7, respectively) compared with that observed in flies expressing *elav>C99^wt^* or *wt* (2.3 ± 0.7 and 0.6 ± 0.4, respectively; Fig. 2*A*,*B*). These results are consistent with previously reported data (Finelli et al., 2004; Iijima et al., 2004, 2008; Chakraborty et al., 2011; Prüßing et al., 2013). We also examined memory performance using a conditioned courtship suppression paradigm (Siegel and Hall, 1979; Kamyshev et al., 1999; Griffith and Ejima, 2009). CI is the fraction of time a male spends in courtship behavior during the observation period. Kruskal–Wallis ANOVA test did not show any significant difference among naive males from all experimental groups [H: (3, *N* = 104) = 2.39 *p *=* *0.5014], demonstrating that the sexual activity of these males was equal. Both *elav>C99^wt^* and *elav>C99^V717I^* as well as *elav>Aβ42* males showed no significant decrease in courtship activity compared with their naive counterparts (*elav>C99^wt^* CI_naive_ = 33.133 vs CI_trained_ = 17.194 *U *=* *196.5, *p *=* *0.0891; *elav>*C99*^V717I^* CI_naive_ = 32.650 vs CI_trained_ = 14.189, *U *= 175, *p *=* *0.0504; *elav>AB42* CI_naive_ = 38.889 vs CI_trained_ = 29.487 *U *= 333.5, *p *=* *0.1252), while *elav>w*^1118^ driver-control males showed a significant decrease in courtship activity (*elav>w*^1118^ CI_naive_ = 33.340 vs CI_trained_ = 3.704, *U *=* *130, *p *<* *0.0001; Fig. 2*C*).

Since all tests for trained males were done in the span of 30 min after a 1-h training session, it can be defined as a test for STM performance (Kamyshev et al., 1999; McBride et al., 2005). The difference between CIs of trained and naive males can be represented as a MI (Kamyshev et al., 1999; Lim et al., 2018). Kruskal–Wallis ANOVA test revealed significant differences in memory performance between driver control line and transgenic lines [H: (3, *N* = 107) = 19.09, *p* < 0.001]. We found that males expressing *elav>C99^V717I^* and *elav>Aβ42* transgenes showed a significant loss in STM compared with *elav>w*^1118^ driver control line (*p *<* *0.05 and *p *<* *0.001, respectively). However, it has to be noted that males expressing wild-type C99 also exhibited a reduction in STM performance, although this difference was not statistically significant (Fig. 2*D*).

Together, these data suggest that expression of *Aβ42* either in fly eyes (*gmr-GAL4*) or pan-neuronally (*elav-GAL4*) produced the most pathologic phenotypes while expression of the London mutation C99*^V717I^* generally produced more severe phenotypes compared with wild-type C99. Thus, our results support previous findings (Finelli et al., 2004; Iijima et al., 2004, 2008; Chakraborty et al., 2011; Prüßing et al., 2013) and provide us with models to evaluate the effect of RAS inhibitors on the development of AD-related phenotypes.

### Captopril and losartan suppress degenerative phenotypes observed in mutant *C99^V717I^* and *Aβ42* flies

**Figure 3.**
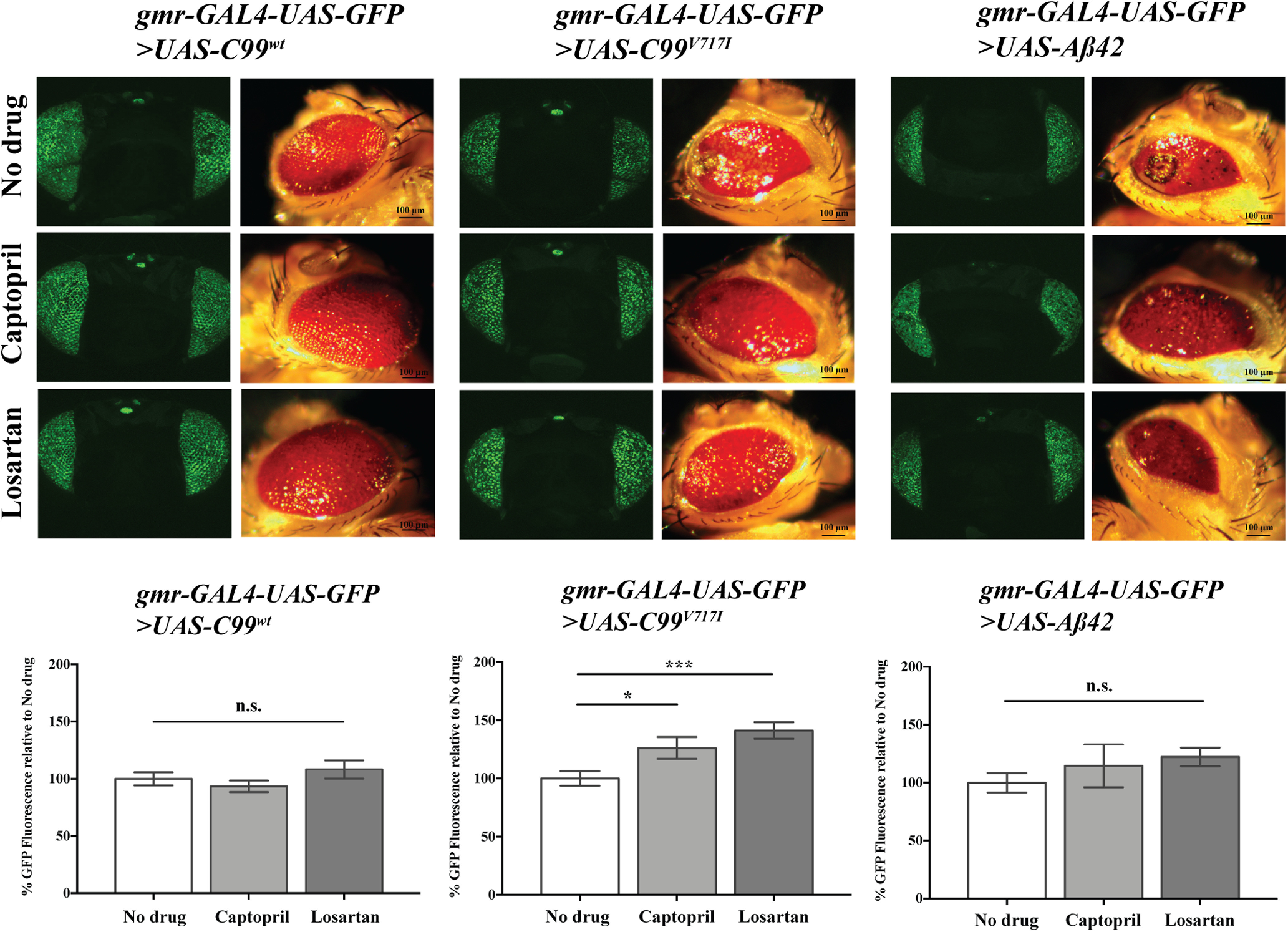
Captopril and losartan increase retinal GFP in flies expressing AD London Mutation, *C99^V717I^.* Confocal GFP and light microscope images of 7-d *gmr-GAL4-UAS-GFP>UAS-C99^wt^*, *gmr-GAL4-UAS-GFP>UAS-C99^V717I^*, and *gmr-GAL4-UAS-GFP>UAS-Aβ42* fly heads shown as labeled with or without drug treatments (top panel). One-way ANOVA of GFP quantification in *gmr>C99^wt^* flies showed no significant differences when administered either drug (*N *=* *49 for captopril; *N *=* *34 for losartan, *p *=* *0.2374). Similar results were found for *gmr>Aβ42* flies (*N *=* *25 for captopril; *N *=* *28 for losartan, *p *=* *0.182). However, one-way ANOVA of GFP quantification in *gmr>C99^V717I^* flies showed a significant effect of drug condition (*p *=* *0.0006). *Post hoc* analysis using Bonferroni’s multiple comparison test showed that both captopril (*N *=* *51) and losartan (*N *=* *61) significantly increased retinal GFP (*p *=* *0.0363, *p *=* *0.0003, respectively). Data are shown as mean ± SEM; *p <* *0.05, ***p <* *0.001; n.s., not significant.

**Figure 4.**
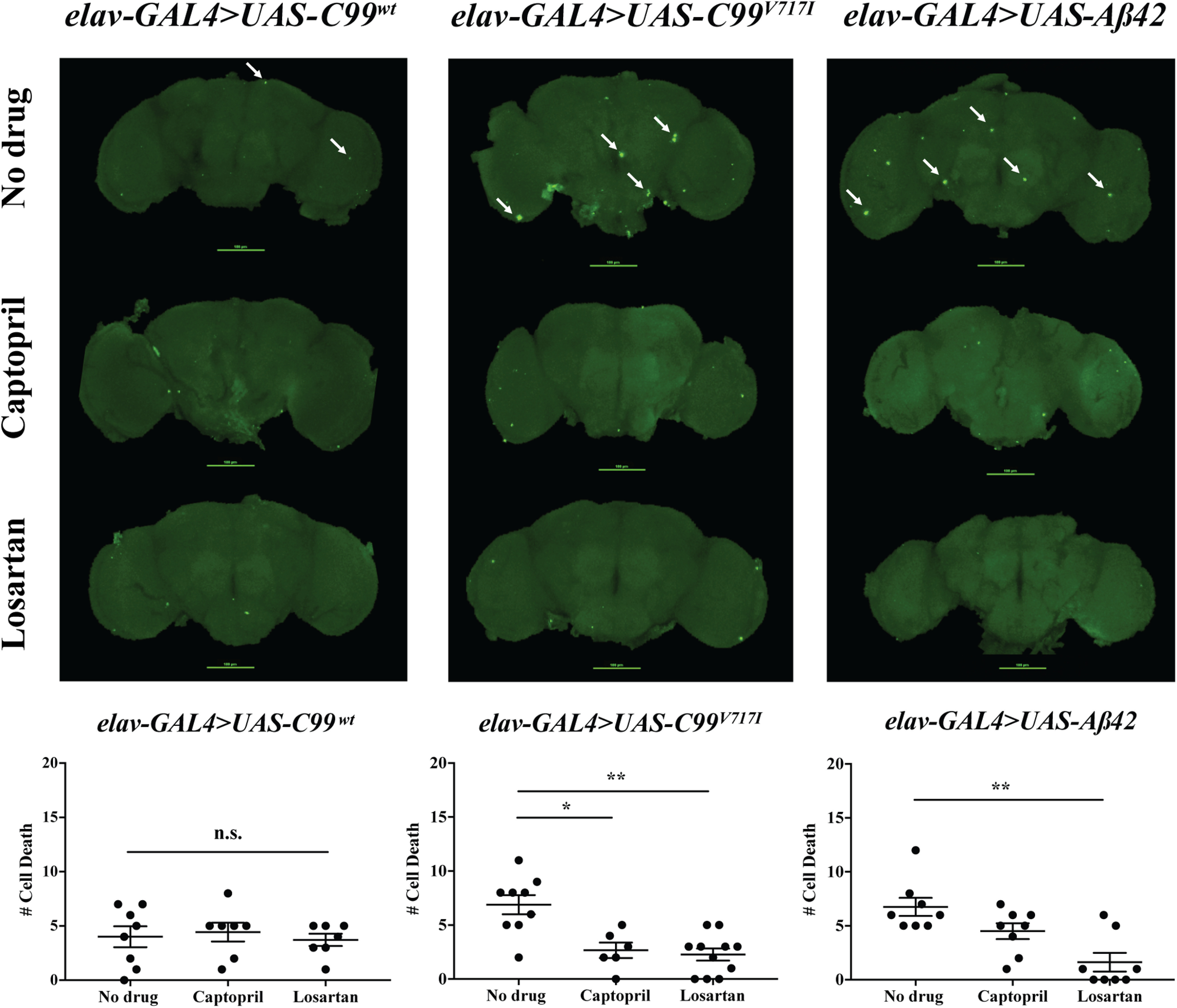
Captopril and losartan reduce TUNEL-labeled brain cell death in flies expressing AD London Mutation, *C99^V717I^*and *Aβ42*. Confocal microscope images of 28-d *elav-GAL4>UAS-C99^wt^*, *elav-GAL4>UAS-C99^V717I^*, and *elav-GAL4>UAS-Aβ42* fly brains with or without drug treatments are shown as labeled. Kruskal–Wallis ANOVA analysis showed that flies expressing *C99^wt^*(*N *≥* *7 per condition) had no significant difference in the number of cell death when compared between no drug versus drugs (*p *=* *0.768). However, Kruskal–Wallis analysis with Dunn’s multiple comparisons test showed that flies expressing *C99^V717I^* (*N *≥* *6 per condition) had significant lower number of cell death in drug-treated flies when compared between captopril to no drug and losartan to no drug (*p *=* *0.0343 and *p *=* *0.0035, respectively). Similarly, for flies expressing *Aβ42* (*N *≥* *8 per condition), a significant lower number of cell death was observed in losartan-treated flies when compared with no drug (*p *=* *0.0066). Data are shown as mean ± SEM; *p <* *0.05, **p <* *0.01; n.s., not significant.

To determine whether captopril or losartan could suppress the rough eye phenotype observed in *Drosophila* expressing AD-related transgenes, we raised flies on medium with and without drugs and examined GFP intensity as described (Fig. 1). We did not observe any effect of either drug on GFP intensity in flies expressing *C99^wt^* or *Aβ42* (Fig. 3). In contrast, *gmr>C99^V717I^* flies exhibited significant increases in retinal GFP expression (26% and 41%, respectively) after administration of either captopril or losartan. Similarly, both drugs significantly reduced the number of TUNEL-labeled brain cells in four-week-old *elav>C99^V717I^* flies (Fig. 4). Moreover, a similar effect was observed in *elav>Aβ42* flies that were fed with losartan for 28 d, whereas *elav>C99^wt^* flies showed no differences in TUNEL-labeled brain cells regardless of drug condition (Fig. 4).

Together, these data demonstrate that known inhibitors of the RAS signaling pathway in humans (captopril and losartan) can suppress toxic phenotypes observed in the eye and CNS of flies expressing AD-related transgenes.

### Captopril and losartan selectively rescue STM in mutant *C99^V717I^* and *Aβ42* flies

**Figure 5.**
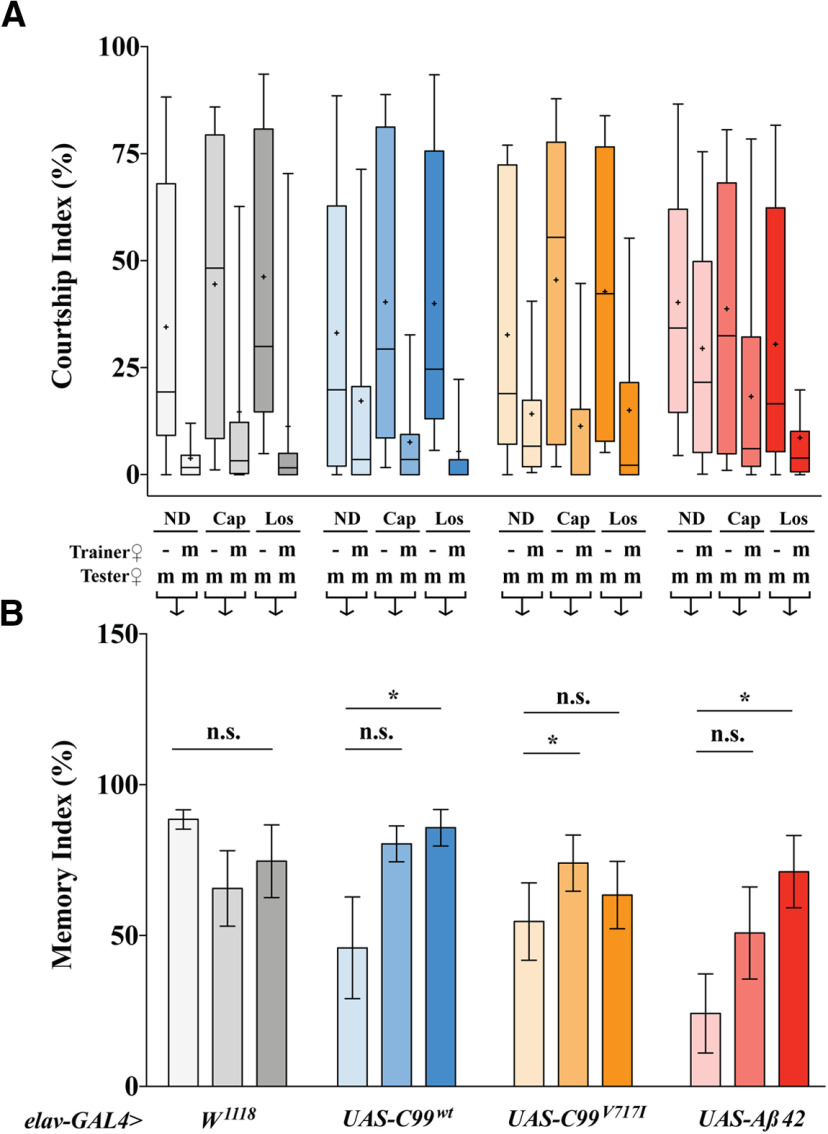
Captopril and losartan selectively rescue STM in *elav>C99^V717I^* and *elav>Aβ42 flies*. ***A***, Percentage of CIs. CIs were calculated by dividing the time a male spent in courtship to a total given time. Trainer and tester females: –, none; m, mated female. Box-and-whisker plots for CI show 10th, 25th, 75th, and 90th percentiles and mean (+). ***B***, Percentage of MIs. MIs were calculated as [100 [1 – (CI with training/mean of CI without training)]. Kruskal–Wallis test followed by Dunn’s multiple comparisons test were used for statistical comparisons (*N ≥ *20 per genotype per condition). *elav-GAL4>w^1118^* flies showed no significant difference in MIs when compared no drug to captopril (*p *=* *0.5171) and losartan (*p *>* *0.9999) conditions. *elav-GAL4>UAS-C99^wt^*flies showed no significant difference in MIs when compared no drug to captopril (*p *=* *0.5171) but losartan (*p *=* *0.0436). *elav-GAL4>UAS-C99^V717I^* flies showed statistically significant MIs when compared no drug to captopril (*p *=* *0.0271) but losartan conditions (*p *=* *0.333). *elav-GAL4>UAS-Aβ42 flies* showed no significant difference in MIs when compared no drug to captopril (*p *=* *0.2459) but losartan (*p *=* *0.045). Data are shown as mean ± SEM; *p <* *0.05; n.s., not significant.

To determine whether captopril or losartan could restore cognitive function in our AD models we examined STM using the courtship conditioning paradigm described in Figure 2 (Siegel and Hall, 1979; Kamyshev et al., 1999). Since lack of courtship activity in naive males may significantly skew the results of courtship conditioning, we first analyzed the potential differences in male sexual activity among naive males of different genotypes and drug conditions. A two-way ANOVA did not reveal any significant effects for genotype (*F*_(3,272)_ = 0.624, *p *=* *0.599), drug condition (*F*_(2,272)_ = 0.577, *p *=* *0.563), or their interaction (*F*_(6,272)_ = 0.668, *p *=* *0.596). Courtship and memory indices for all comparable groups are shown (Fig. 5; note that we have also included the data from Fig. 2 for “no drug” condition for comparative purposes). We found that administration of either drug (captopril or losartan) did not significantly change 30-min STM in the *elav>w^1118^* control flies (Fig. 5), whereas for the transgenic lines these drugs exert a selective effect. Administration of both drugs in these flies resulted in an increased MI, similar to that observed in *elav>w^1118^* controls. However, because of large variance within the *elav>C99^wt^* expressing flies, the multiple comparison test revealed statistical significance only for losartan. Opposite effect was observed in flies expressing *elav>C99^V717I^*, captopril shows a significant memory improvement while losartan does not. *elav>Aβ42* flies showed obvious increase of MI in response to both drugs, althouth only for losartan the effect was statistically significant (Fig. 5). Overall, these data demonstrate that known inhibitors of the RAS pathway in humans, can significantly improve memory performance in *Drosophila* expressing AD-related transgenes.

### Captopril and losartan do not suppress degenerative phenotypes observed in *Tau* flies

**Figure 6.**
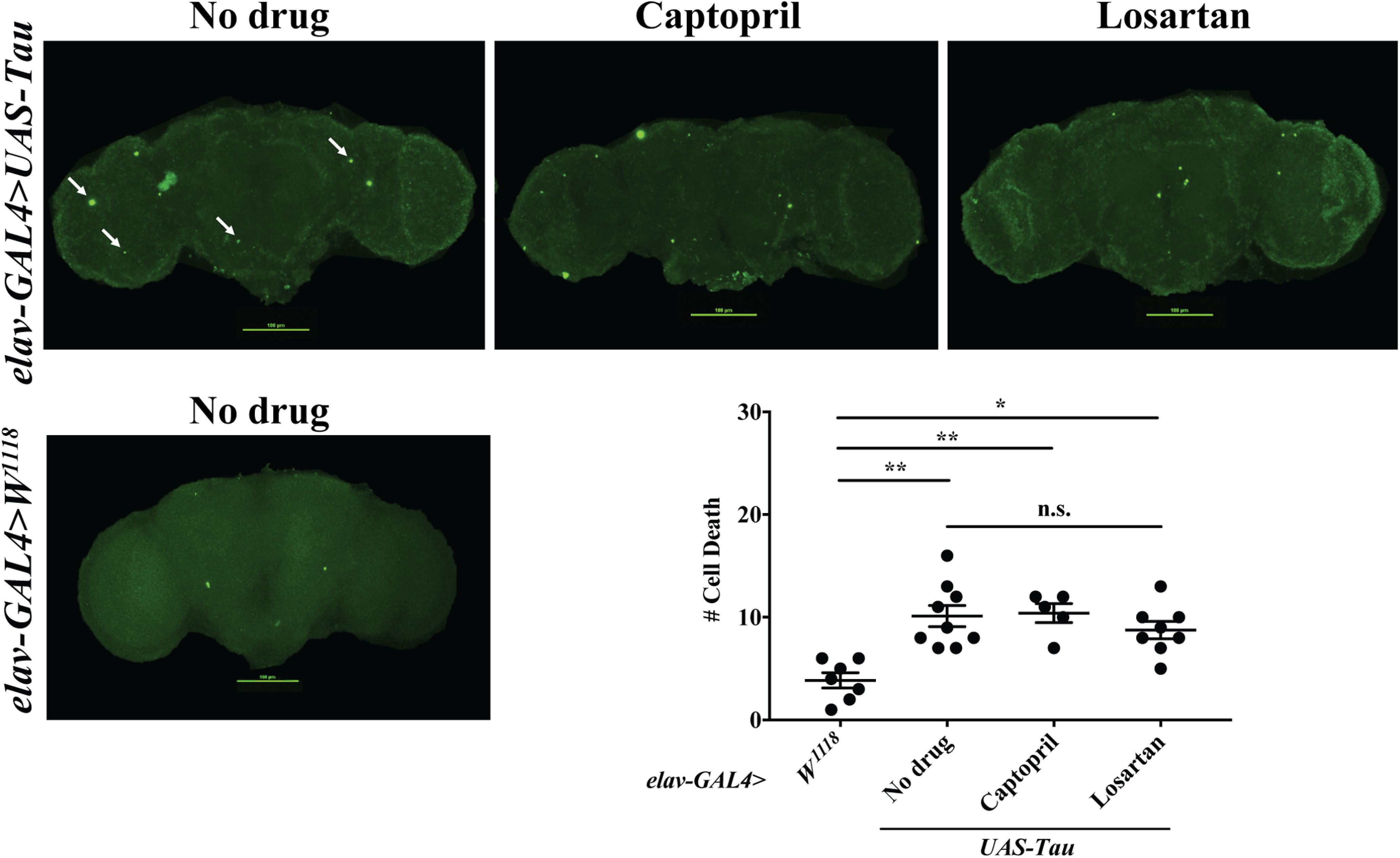
Captopril and losartan do not affect number of TUNEL-labeled brain cell death in flies expressing *Tau*. TUNEL labeling in brains of 28-d-old flies are shown as labeled. Kruskal–Wallis ANOVA analysis with Dunn’s multiple comparisons test showed that flies expressing *Tau* +/− captopril or losartan have a significant higher amount of TUNEL-labeled cell death compared with *wt* (*N ≥ *5 per condition; *p *=* *0.0035, *p *=* *0.0064, and *p *=* *0.0404, respectively). However, no significant change was observed when compared captopril-treated or losartan-treated flies to no drug (*N ≥ *5 per condition; *p *>* *0.9999 and *p *>* *0.9999, respectively). Data are shown as mean +/-SEM; **p < 0.05, ***p <* *0.01; n.s., not significant.

To determine whether captopril and losartan exert beneficial effects in other forms of AD, we examined their ability to suppress brain cell death in flies expressing human Tau protein. Previous studies have shown expression of human Tau in animal models leads to several neurodegenerative phenotypes similar to human AD cases including an increase in cell death, shrinkage in brain size and defects in cognitive ability (Wittmann et al., 2001; Gistelinck et al., 2012). We found that neither drug affected the number of TUNEL-labeled brain cells when maintained on either captopril or losartan for 28 d in *elav>Tau* flies (Fig. 6), suggesting that the beneficial effects of RAS inhibitors are specific to APP-CTF and Aβ42 expressing flies.

### Captopril and losartan do not affect APP-CTF or *Aβ42*

**Figure 7.**
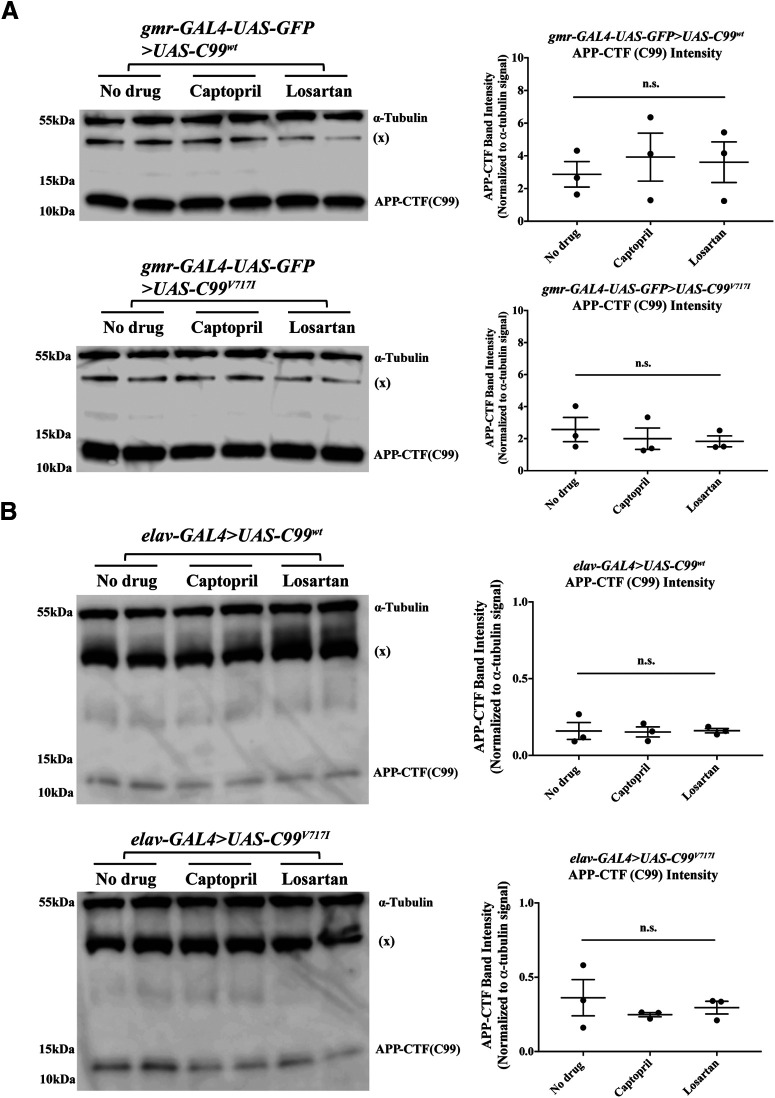
Captopril and losartan do not change C99 levels in either *gmr* or *elav* model of C99 expressing flies. ***A***, Western blottings using samples from *gmr-GAL4-UAS-GFP>UAS-C99^wt^* and *gmr-GAL4-UAS-GFP>UAS-C99^V717I^*heads with or without drug treatments are shown as labeled. Each condition was tested with two technical replicates each time with a total of three biological replicates (*N *=* *3, *n *=* *2). Kruskal–Wallis ANOVA analysis showed that both captopril and losartan had no significant effects on the levels of C99 in both *gmr>C99^wt^* and *gmr>C99^V717I^* flies at 7 d (*p *=* *0.9929 and *p *=* *0.5429, respectively). ***B***, Western blottinigs using samples from *elav-GAL4>UAS-C99^wt^* and *elav-GAL4>UAS-C99^V717I^*heads with or without drug treatments are shown as labeled. Each condition was tested with two technical replicates each time with a total of three biological replicates (*N *=* *3, *n *=* *2). Kruskal–Wallis ANOVA analysis showed that both captopril and losartan had no significant effects on the levels of C99 in both *elav>C99^wt^* and *elav>C99^V717I^* flies at 28 d (*p *=* *0.8786 and *p *=* *0.7214, respectively). Data are shown as mean ± SEM; n.s., not significant.

Previous studies have suggested that ACE-Is may be beneficial in AD by regulating the production, degradation, conversion and/or clearance of Aβ peptides. Whether ARBs have similar effects is unknown. To determine whether the beneficial effects of RAS inhibitors on brain cell neurodegeneration and STM in our AD-related transgenic flies occur through similar mechanisms we first used Western blotting to quantitate the levels of C99 in the presence or absence of drugs. We found that administration of either captopril or losartan throughout the adult lifespan of both *C99^wt^* and mutant *C99^V717I^* flies had no effects on the levels of C99 in either fly eyes (*gmr-GAL4* driver) or in the central nervous system (*elav-GAL4* driver; Fig. 7*A*,*B*, respectively).

**Figure 8.**
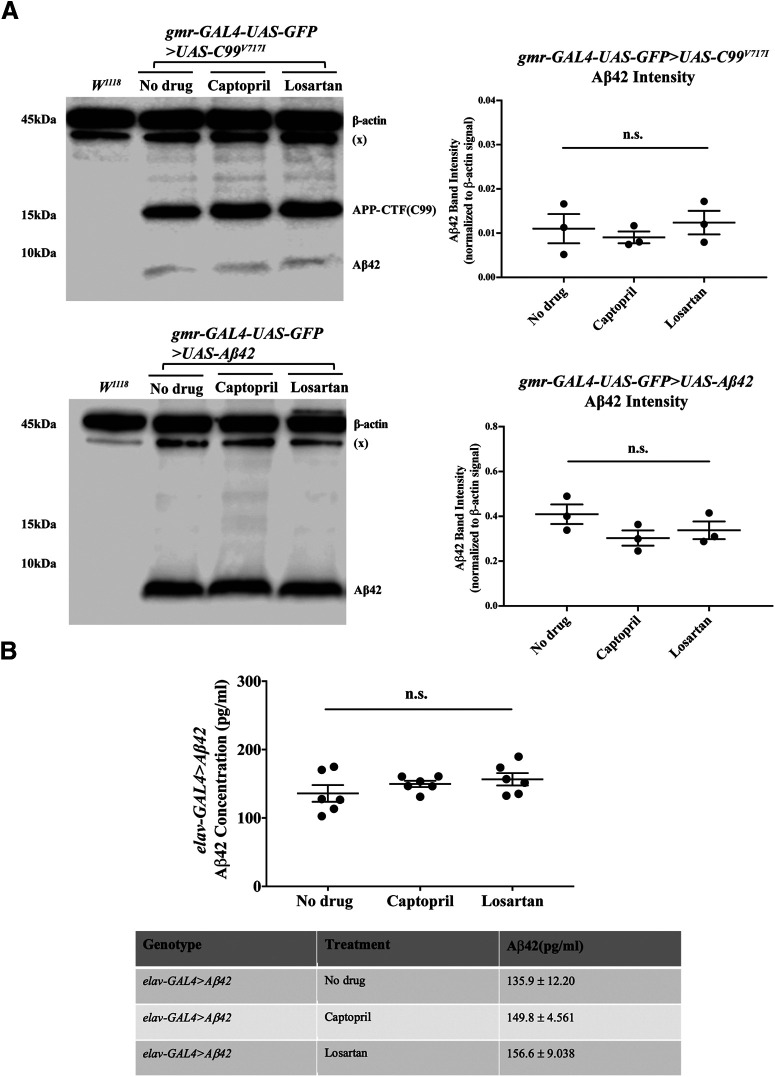
Captopril and losartan do not change soluble Aβ42 levels in flies expressing Aβ42 using a *gmr* or *elav* driver. ***A***, Western blottings using samples from *gmr-GAL4-UAS-GFP>UAS-C99^V717I^* and *gmr-GAL4-UAS-GFP>UAS-Aβ42* heads with or without drug treatments are shown as labeled. Each condition was tested with three biological replicates (*N = 3*). Kruskal–Wallis ANOVA analysis showed that both captopril and losartan had no significant effects on the levels of soluble Aβ42 in both *gmr>C99^V717I^* and *gmr>Aβ42* flies at 7 d (*p *=* *0.6286 and *p *=* *0.2964, respectively). ***B***, Levels of Aβ42 in *elav-GAL4>UAS-Aβ42* heads at 28 d after eclosion were measured using human Aβ42 ELISA. The two-tailed unpaired *t* test showed that captopril had no significant effect on Aβ42 levels when compared with no drug condition (*p *=* *0.31). A similar result was observed in *elav-GAL4>UAS-Aβ42* flies treated with losartan (*p *=* *0.5182). Each condition was tested with three technical replicates and two biological replicates in total (*N *=* *2, *n *=* *3). Data are shown as mean ± SEM; n.s., not significant.

**Figure 9.**
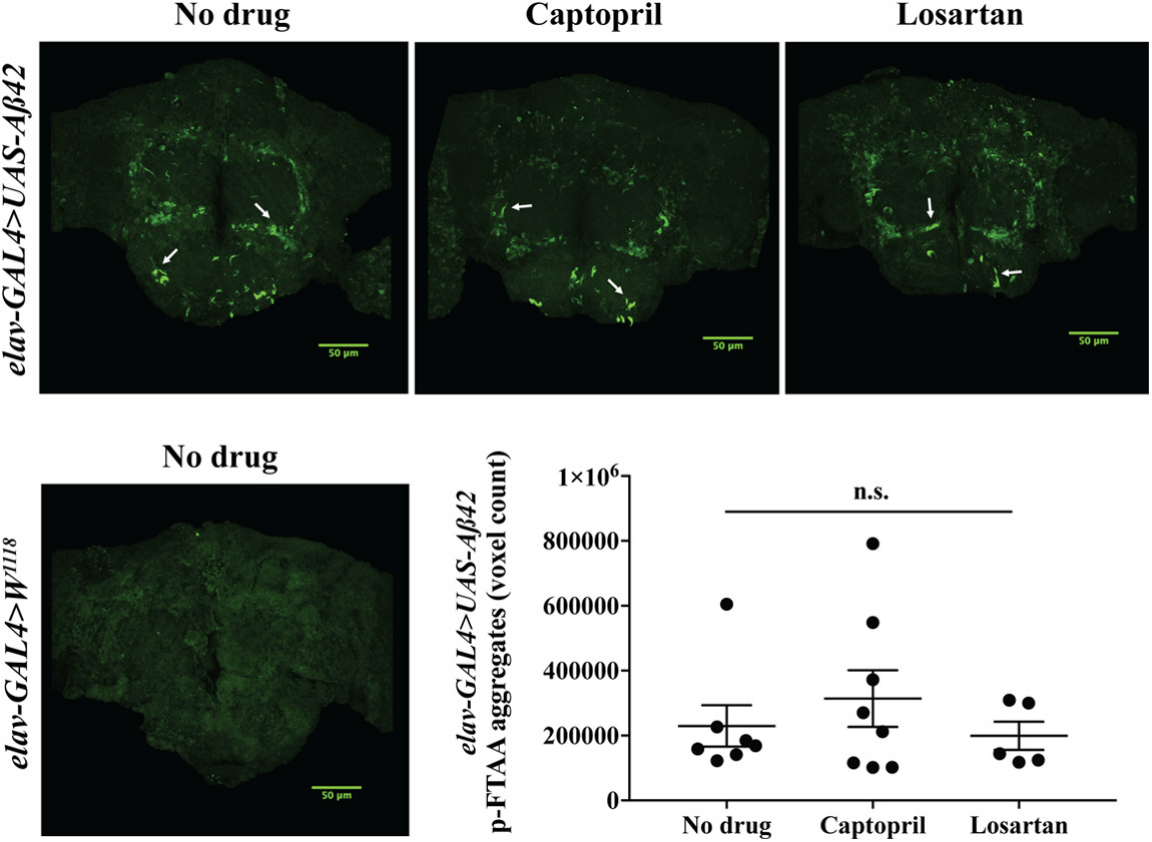
Captopril and losartan do not change Aβ aggregates in *elav>Aβ42* flies. Whole *Drosophila* brain staining with the amyloid-specific LCO, p-FTAA (green) in *elav-GAL4>W^1118^*, and *elav-GAL4>UAS-Aβ42* flies are shown as labeled. Staining reveal Aβ aggregates in *elav-GAL4>UAS-Aβ42* flies (white arrows). Quantification and comparison of Aβ aggregates (p-FTAA pixels) in *elav-GAL4>UAS-Aβ42* flies with or without drug treatment at 28 d after eclosion using Kruskal–Wallis ANOVA analysis revealed no significant changes (*p *=* *0.9516; *N *≥* *5 per condition). Data are shown as mean ± SEM; n.s., not significant.

We then asked whether captopril or losartan affect the levels of Aβ peptides by measuring the soluble Aβ42 levels from lysates of adult fly heads using Western blotting and ELISA. We found that administration of either captopril or losartan throughout the adult lifespan of *gmr>C99^V717I^* and *gmr*>*Aβ42* flies had no effect on the levels of Aβ42 at 7 d after eclosion (Fig. 8*A*). Similarly, neither drug had significant effect on the levels of Aβ42 in *elav*>*Aβ42* flies at 28 d after eclosion (Fig. 8*B*); Aβ42 was undetected in both *elav>C99^wt^* and mutant *elav>C99^V717I^* regardless of drug treatment. To examine the effects of both drugs on insoluble Aβ42, we measured and compared Aβ aggregates in the brains of *elav>Aβ42* flies with or without drug treatment using the amyloid-specific LCO, p-FTAA stain, to detect Aβ plaques at 28 d after eclosion. Comparison across different conditions revealed no significant changes (Fig. 9). Together, these results suggest that the beneficial effects of captopril and losartan are independent of APP-CTF processing or accumulation/clearance of Aβ42.

### A null mutation in *Drosophila Acer* recapitulates the beneficial effects of captopril in *C99^V717I^* and *Aβ42* flies

**Figure 10.**
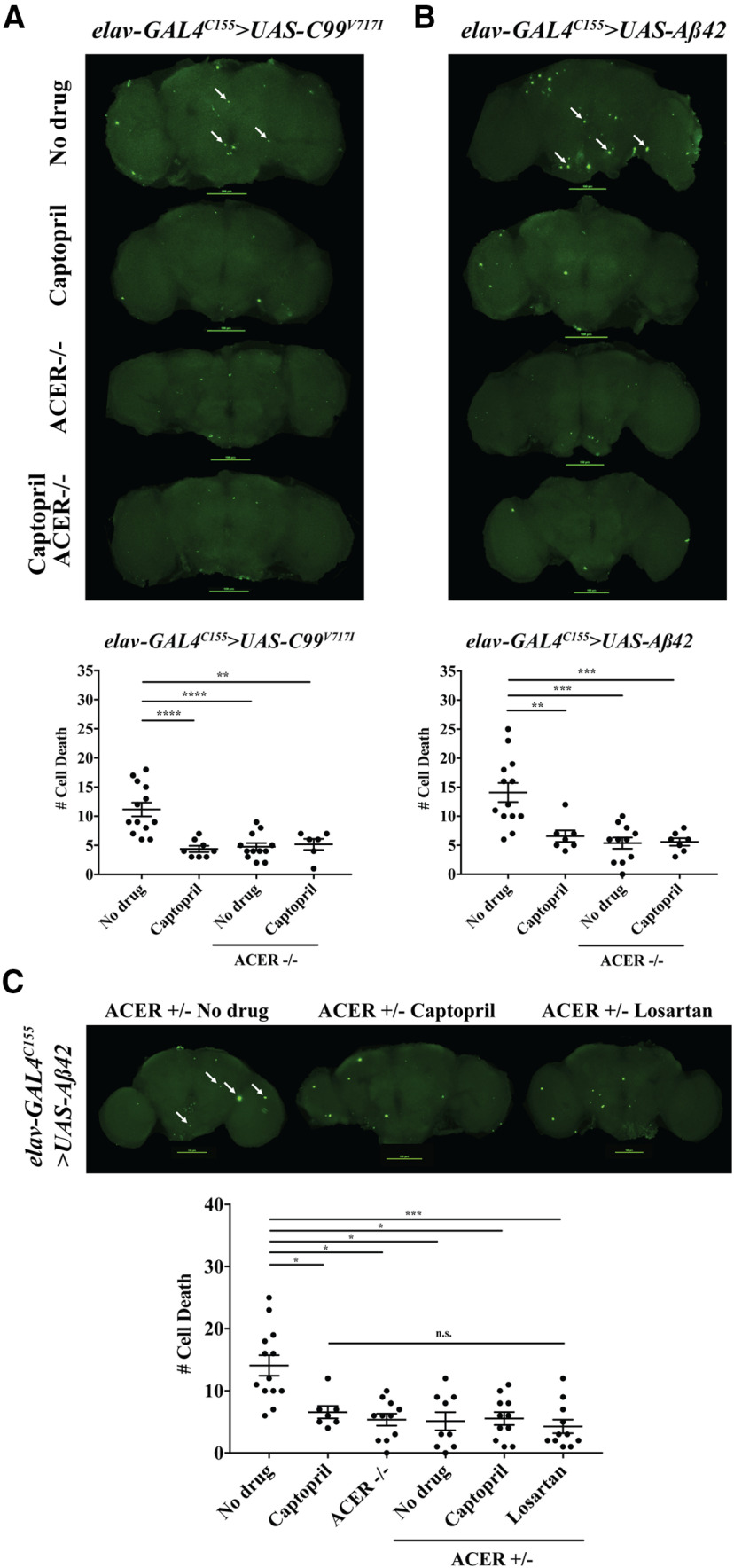
A homozygous *Acer* null mutant reduces brain cell death in flies expressing *C99^V717I^*and *Aβ42*. Confocal microscope images of 28-d (***A***) *elav-GAL4^C155^>UAS-C99^V717I^* and (***B***) *elav-GAL4^C155^>UAS-Aβ42* fly brains in the presence or absence of captopril and an *Acer* null mutation are shown as labeled. Mann–Whitney analysis showed that *C99^V717I^*flies (*N *≥* *6 per condition) treated with captopril as well as those carrying an *Acer* null mutant +/− captopril had significantly lower numbers of cell death than compared with control flies on no drug (*p *<* *0.0001, *p *<* *0.0001, and *p *=* *0.0031, respectively). A similar effect was observed in *Aβ42* flies (*N *≥* *7 per condition) treated with captopril or in flies carrying an *Acer* null mutations +/− captopril (*p *=* *0.003, *p *=* *0.0001, and *p *=* *0.0004, respectively). ***C***, *elav-GAL4^C155^>UAS-Aβ42* fly brains with an *Acer* heterozygous null mutation in the presence or absence of captopril and losartan are shown as labeled (*N *≥* *9 per condition). Kruskal–Wallis ANOVA analysis with Dunn’s multiple comparisons test showed that an *Acer* heterozygous null mutant had significantly lower numbers of cell death compared with *elav^C155^> Aβ42* flies on no drug (*p *=* *0.0156). No significant difference was found when compared with either plus captopril or losartan or an *Acer* homozygous null mutant (*p *>* *0.9999 for all comparisons). Data are shown as mean ± SEM; *p <* *0.05, **p <* *0.01, ***p <* *0.001, ****p <* *0.0001; n.s., not significant.

**Figure 11.**
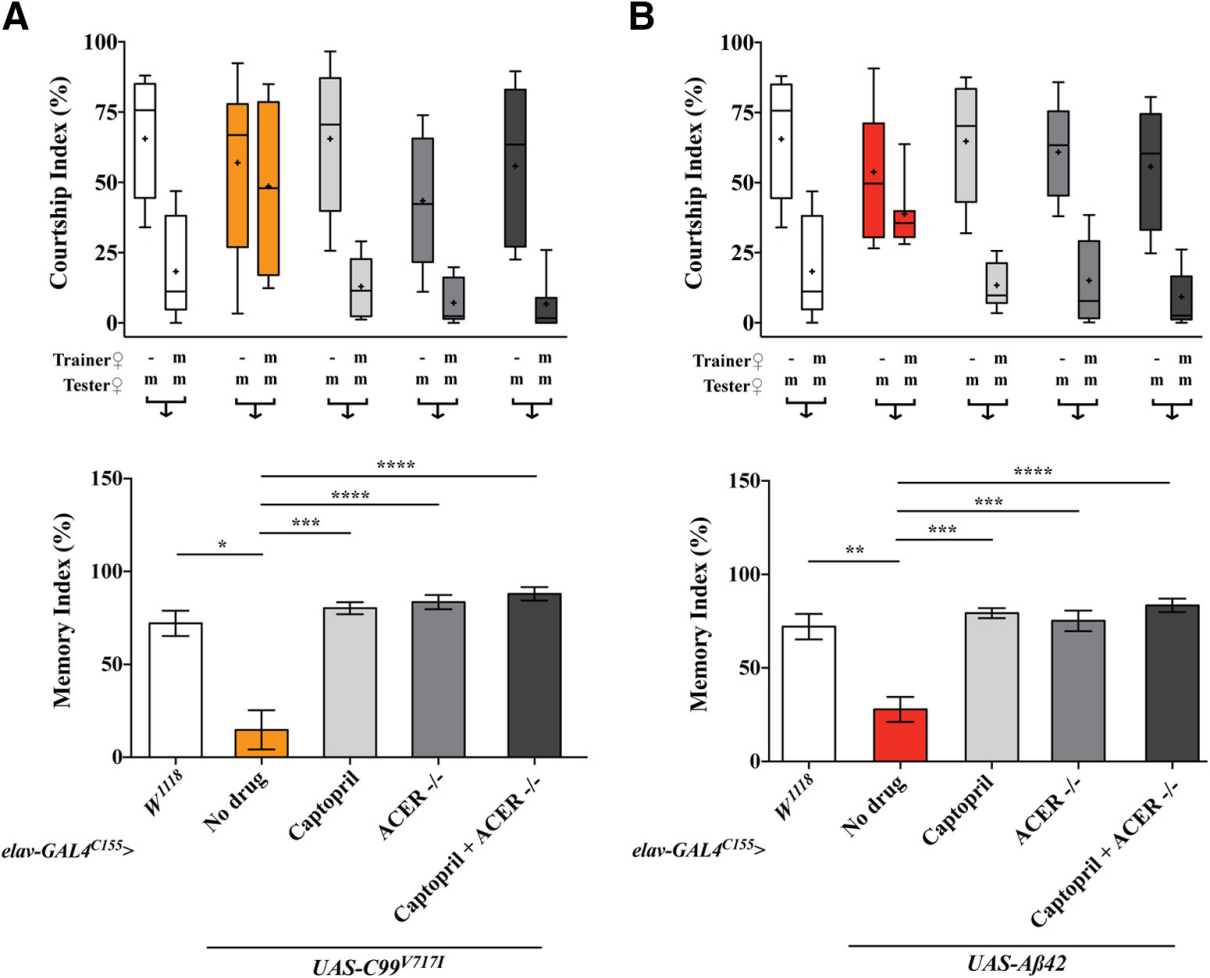
A homozygous *Acer* null mutant rescues STM in flies expressing *C99^V717I^*and *Aβ42*. Percentage of CIs and MIs are shown as labeled for (***A***) *elav-GAL4^C155^>UAS-C99^V717I^* and (***B***) *elav-GAL4^C155^>UAS-Aβ42* flies. CIs were calculated by dividing the time a male spent in courtship to a total given time. Trainer and tester females: –, none; m, mated female. Box-and-whisker plots for CI show 10th, 25th, 75th, and 90th percentiles and mean (+). MIs were calculated as [100 [1 – (CI with training/mean of CI without training)]. Kruskal–Wallis test followed by Dunn’s multiple comparisons test was used for statistical comparisons (*N ≥ *20 per genotype per condition). *elav-GAL4^C155^>UAS-C99^V717I^* flies treated with captopril as well as those carrying an *Acer* null mutant +/− captopril had significantly higher MIs when compared with no drug condition (*p *=* *0.0005, *p *<* *0.0001, and *p *<* *0.0001, respectively). A similar effect was observed in *Aβ42* flies treated with captopril or in flies carrying an *Acer* null mutant +/− captopril (*p *=* *0.0001, *p *<* *0.0001, and *p *=* *0.0001, respectively). Data are shown as mean ± SEM; *p <* *0.05, **p <* *0.01, ***p <* *0.001, ****p <* *0.0001; n.s., not significant.

To determine whether components of RAS underlie the beneficial effects of captopril (ACE-I) in our *Drosophila* AD models, we obtained an *Acer* null mutant (Carhan et al., 2011) and recombined it with our AD transgenic lines *elav-GAL4^C155^>UAS-C99^V717I^* or *elav-GAL4^C155^>Aβ42. elav-GAL4^C155^*driver was used instead of *elav-GAL4/CyO* for genetic recombination purposes and generated flies expressing *C99^V717I^* or *Aβ42* in a homozygous *Acer* null background. Since *elav-GAL4^C155^* endogenously drives expression of GAL4 at higher levels, the phenotypes observed in our transgenic lines were more severe than those previously observed using *elav-GAL4/CyO*, which expresses GAL4 at lower levels. Of note, although there are several ACE homologs in *Drosophila*, we focused on *Acer* since previous studies have shown that it contains the N-terminal catalytic site observed in human ACE and can be inhibited by captopril *in vitro* (Houard et al., 1998). We found that a null mutation in *Acer* significantly reduced brain cell death in both four-week-old *elav^C155^>C99^V717I^* and *elav^C155^> Aβ42* flies similar to what we observed after captopril treatment (Fig. 10*A*,*B*). Similarly, an *Acer* null mutation also rescued STM in both four-week-old *elav^C155^>C99^V717I^* and *elav^C155^> Aβ42* flies (*p *<* *0.0001, *p *=* *0.0001, respectively, compared with no drug treatment; Fig. 11). Importantly, we did not observe any additive effects when the same flies were fed captopril for 28 d after eclosion (Figs. 10*A*,*B*, 11). Interestingly, we also observed that flies heterozygous for the *Acer* null mutation also suppressed brain cell death in four-week-old *elav^C155^> Aβ42* flies similar to captopril treatment and no additive effects were found when fed with either captopril or losartan (Fig. 10*C*). Together, these data are consistent with Acer being the target of captopril that mediates the beneficial effects observed in our transgenic lines expressing AD-related transgenes. Whether losartan acts in the same downstream pathway remains to be determined and requires further targets to be discovered.

## Discussion

Recent studies have shown that administration of antihypertensive medications such as ACE-Is and ARBs are associated with reduced onset and progression of AD. However, the mechanisms by which these drugs lead to beneficial effects in AD are unclear. Here, we examined the effects of captopril (ACE-I) and losartan (ARB) in *Drosophila* that express human AD-related transgenes in the eye and CNS. We found that administration of either drug significantly reduced cell death within the brain and improved STM. We also found that the beneficial effects were most pronounced in flies expressing Aβ42 peptides although neither drug affected the production, accumulation or clearance of Aβ42. We also observed no effects of either drug on degenerative phenotypes in *Drosophila* expressing human Tau, suggesting that the beneficial effects are specific to APP-CTF and Aβ42 expressing flies. Finally, we found that the beneficial effects observed on captopril treatment could be completely recapitulated by introducing an *Acer* null mutation into our AD fly models consistent with Acer being the target of captopril in *Drosophila*. Interestingly, while ACE orthologs have been identified in *Drosophila* the RAS, which includes downstream effectors of ACE, including angiotensin I/II and the angiotensin receptor, are not conserved. This suggests that the beneficial effects of ACE-Is and ARBs in *Drosophila* may involve mechanisms that are distinct from those mediated by the canonical RAS.

Several studies have shown that use of ACE-Is and ARBs correlates with decreased incidence and improved cognitive outcomes in AD patients (Ohrui et al., 2004; Davies et al., 2011; Qiu et al., 2013; Yasar et al., 2013; de Oliveira et al., 2014; Wharton et al., 2015; Ho et al., 2017). Importantly, only brain-penetrating ACE-Is and ARBs have been shown to delay the onset of cognitive impairment and neurodegeneration in mice models and humans, demonstrating that their beneficial effects are independent of their role in regulating blood pressure (Alvarez et al., 1999; Braszko et al., 2003; Ohrui et al., 2004; Hajjar et al., 2005; Edwards et al., 2009; Miners et al., 2009; Belbin et al., 2011; Davies et al., 2011; Gao et al., 2013; Qiu et al., 2013; Soto et al., 2013; Yasar et al., 2013; de Oliveira et al., 2014; Kauwe et al., 2014; O’Caoimh et al., 2014; Wharton et al., 2015; Ho et al., 2017). Several *in vitro* studies have suggested that ACE may be involved in Aβ degradation, conversion, and clearance (Kehoe et al., 1999; Hemming and Selkoe, 2005; Liu et al., 2014). *In vivo* studies, however, are controversial with some studies demonstrating that ACE-Is promote Aβ42 deposition (Zou et al., 2007; Bernstein et al., 2014), have little to no effect on Aβ42 peptide levels or plaque deposition (Hemming et al., 2007; Dong et al., 2011), and reduce Aβ deposits in the hippocampus (Abdalla et al., 2013). Despite this conflicting evidence, ACE-Is have consistently demonstrated improved cognitive outcomes in mice models of AD and in patients (Ohrui et al., 2004; Hajjar et al., 2005; El Sayed et al., 2009; Yamada et al., 2010; Dong et al., 2011; AbdAlla et al., 2013; Soto et al., 2013; de Oliveira et al., 2014; O’Caoimh et al., 2014). Similarly, ARBs have also been reported to improve cognitive function in rodent models (Takeda et al., 2009; Tsukuda et al., 2009; Shindo et al., 2012; Bild et al., 2013; Singh et al., 2013; Royea et al., 2017) but do not appear to alter Aβ levels (Ongali et al., 2014) or aggregation (Ferrington et al., 2011).

Given the known role of ACE-Is and ARBs in modulating RAS, several *in vivo* studies have examined the effect of regulating specific components of RAS on AD related phenotypes. These studies demonstrated toxic effects associated with Ang II/AT1R signaling in the brain resulting in an increase in the levels and deposition of Aβ42 (Faraco et al., 2016), increased oxidative stress and enhanced cognitive defects (Bild et al., 2013; Royea et al., 2017). On the other hand, protective effects including a decrease in neuronal degeneration and improved cognitive function, were observed with enhanced Ang II/AT2R and Ang IV/AT4R signaling (Bild et al., 2013; Royea et al., 2017). In line with these findings, studies have also shown beneficial roles of ACE-Is and ARBs in animal models of AD whereby the drugs prevented Ang II production and inhibited Ang II/AT1R signaling (Tsukuda et al., 2009; AbdAlla et al., 2013; Royea et al., 2017). Together, these studies suggest that the protective effects of ACE-Is and ARBs in AD may be associated with inhibition of Ang II/AT1R signaling, however, the role of RAS in AD pathology is still unclear.

We first identified two ACE-like factors in *Drosophila*, *Acer* and *Ance-5*, in a genetic screen for PS and C99 modifiers (van de Hoef et al., 2009). Interestingly, although *Drosophila* have ACE orthologs, the canonical RAS that includes angiotensin I/II and the angiotensin receptor is not conserved. Importantly, only *Acer* and *Ance-5* were identified in our screen and, of these, Acer shares greater amino acid similarity and identity to human ACE and also retains the ACE active site and enzyme activity (Coates et al., 2000). In addition, ACE inhibitors are significantly more potent toward Acer (Cornell et al., 1995; Houard et al., 1998). Indeed, we found that ACE-Is can significantly reduce cell death within the brain and improve STM in *Drosophila* expressing AD-related transgenes except *Tau*. Moreover, we observed similar beneficial effects in *Drosophila* treated with an ARB, although the angiotensin receptor is not conserved. At present, the mechanism by which ACE-Is and ARBs function in *Drosophila* is unclear. Both captopril and losartan consistently suppress AD-related phenotypes in flies expressing either human C99 carrying a London mutation or Aβ42 however, these beneficial effects are not associated with any changes in the production, accumulation or clearance of Aβ42. This finding is consistent with previous *in vivo* studies in mice and humans demonstrating that ACE-Is and ARBs improved cognitive function without affecting Aβ levels (Hemming et al., 2007; Wharton et al., 2012) but contrasts with *in vitro* studies, demonstrating that ACE-Is lead to increased Aβ42 production and aggregation (Kehoe et al., 1999; Hemming and Selkoe, 2005; Zou et al., 2007; Liu et al., 2014). Therefore, based on our findings, it is unlikely that these drugs are modulating AD-related phenotypes through γ-secretase cleavage of C99. It is also unlikely that the ability of ACE-Is and ARBs to rescue cell death and cognitive dysfunction in *Drosophila* is because of effects on Angiotensin receptors since, other than ACE, the canonical RAS is not conserved in *Drosophila.* At present, the function of *Acer* in *Drosophila* is not fully understood. Some ACE-like factors have been shown to be affected by ACE-Is including *Acer* and *Ance* (Williams et al., 1996; Houard et al., 1998), however, the targets of either protein have yet to be identified. *Acer* null mutants have also been shown to exhibit disruptions in night-time sleep and sleep fragmentation (Carhan et al., 2011) as well as altered behavioral and metabolic responses to diet (Glover et al., 2019). However, these flies develop normally to adulthood, suggesting that major developmental or signaling pathways have not been affected. Flies lacking *Ance* have also been shown to develop normally without any obvious physiological defects (Kim et al., 2017). Similarly, the target for ARBs in *Drosophila* is currently unknown as no homolog of ATR has been discovered. Together, our data demonstrate that ACE-Is and ARBs can alleviate toxic phenotypes in *Drosophila* expressing human AD transgenes. Since these beneficial effects are observed in the absence of the canonical RAS this also suggests that captopril and losartan may be acting on a more ancestral function of this pathway and have additional targets that can be identified in *Drosophila*.

## Synthesis

Reviewing Editor: Karyn Frick, University of Wisconsin-Milwaukee Decisions are customarily a result of the Reviewing Editor and the peer reviewers coming together and discussing their recommendations until a consensus is reached. When revisions are invited, a fact-based synthesis statement explaining their decision and outlining what is needed to prepare a revision will be listed below. The following reviewer(s) agreed to reveal their identity: Teresa Niccoli. Note: If this manuscript was transferred from JNeurosci and a decision was made to accept the manuscript without peer review, a brief statement to this effect will instead be what is listed below.

This manuscript describes the effect of feeding Angiotensin Converting Enzyme Inhibitor (Captopril) and Angiotensin Receptor Blocker (Losartan), both inhibitors of the renin angiotensin system, to APP-related Drosophila models of Alzheimer’s Disease. The authors show a rescue in a number of behavioural and pathological markers of neurodegeneration, but no change in levels of the toxic peptides expressed. The authors then go on to show that the rescue seen by ACE-I is epistatic to deletion of Acer, suggesting the target is indeed Acer. The results were thought by the reviewers to be very interesting, and pave the way to studies aiming at understanding the mechanisms of action of these two drugs. One reviewer also noted the importance of the work, since such drugs were reported to have beneficial effects in mouse models of AD or in some patients. Another reviewer noted that te paper is very well written and presents a good characterization of the toxicity observed in these flies and a reasonably convincing rescue. Nevertheless, the reviewers noted several important concerns as detailed below.

Major points:

It is unclear how these flies are a model of AD. The strains used either express C99, which is not processed to Aß (the authors could not detect any Aß by ELISA), and the Aß strain used is not one conventionally used in the field, as far as I can tell, and it does not appear to contain a signal peptide required for secretion. This means it is not secreted, as Aß peptides would normally be. The authors are therefore mostly looking at intracellular proteotoxic stress, not a model of Alzheimer’s disease, the manuscript still has value, but the title should reflect this.

On a related note, it is not clear if the effect of these drugs is specific to APP-Abeta related neurodegeneration. Because the authors suggest that these drugs could have unconventional targets in Drosophila, it is possible that these targets are beneficial for any kind of neurodegeneration. In particular, concerning AD, it is of importance to know whether these drugs act at the level of amyloid pathology specifically, or can also act (or not) at the level of Tau pathology. The authors should test the effects of these drugs on other ND Drosophila models (in particular Tau models, which also give a eye neurodegeneration phenotype).

The lifespan analysis should use back-crossed flies, and males and females should not be mixed. Fly lifespans are extremely susceptible to back-ground variation, without back-crossing it is unclear if the authors are looking at the effect of the transgene expressed of backgroud genetic variability. The driver alone and single transgene controls should also be run to account for this. Moreover, male flies lifespans are around 30% shorter than those of females, they should not be plotted together, the authors should plot them side by side, separated by sex. This paper: https://www.ncbi.nlm.nih.gov/pmc/articles/PMC5507281/ provides a very good description of how a lifespan should be set up.

Whether observed drug effects are the consequence of the anti-hypertensive action of these drugs in the brain, or is the consequence of the degradation/cleavage of Abeta, or is due to a new mechanism of action is an open question.  Interestingly, in Drosophila, the renin-angiotensin system is not completely conserved :  1) the homologs of the ACE (Angiotensin A converting Enzyme, which cleaves Angiotensin A to produce the active peptide Angiotensin II) are present (Acer, Ance-5....)  2) there is no Angiotensin peptide and no Angiotensin receptor. Hence the ACE homologues must be involved in the cleavage of different peptides.  Because the authors do get a beneficial effect with Losartan (known as a Angiotensin receptor Blocker in Vertebrates) in Drosophila, they conclude that this drug must have a different target in Drosophila. Whether this target is also present in Vertebrates is an open question that should be addressed.   

Another main concern about the work is 1) either the absence of mechanistic insight apart from the implication of Acer (we even do not know whether Losartan has an effect in acer mutants) 2) or the paucity of control experiments to ascertain the fact that captopril and losartan somehow tackle the APP-Abeta neurodegenerative pathway specifically.  It is acknowledged that the authors do not yet know the targets of these drugs in Drosophila and that their work is very useful because they show the protective effects of these drugs apart from the classical renin angiotensin system. However, to conclude that the effects of these drugs i) tackles the same mechanism, ii) is specific of beta-induced neurodegeneration, more control experiments would be needed. Four related points: 

- It is unclear whether the flies stayed on the same medium for 7 days (or 28 days for some experiments), or whether they were flipped everyday on fresh medium with fresh drugs. In addition, it is not clear why the concentrations of 5mM Captopril and 1mM Losartan chosen.  

- Is there a plateau of the activity of each drug, and any additive effect of these drugs (at the max concentration)? This should give further indication about their targets (same target or different targets). It is not clear at all, in the manuscript, whether the authors think these two drugs act on a common mechanism/target or not, and this should be made clearer.

- Because Losartan is not known to act on ACE, we do not expect to see a loss of Losartan effect in acer mutants (unless it acts upstream of Acer, somehow). The authors do show the effects of acer mutation, and the absence of Captotpril effect in this genetic condition. Do they still see an effect of Losartan in acer mutants? This result is very important to conclude about the underlying mechanisms.

- Can the authors verify that there is no effect of higher dose of captopril in acer mutants?

In Fig 2 and 5. The samples in the courtship analysis are not compared to their relative controls. Error bars are also not shown on the Memory Index for the courtship, as the data is collapsed by using means and therefore the authors are showing n=1.

Minor points:

-In the courtship experiments (both Fig 2E-F and Fig 5A-B) a more valid comparison would be control to experimental condition, not everything to a hypothetical null. This could be done by comparing the CI to each other rather than use MI for the statistical analysis. The comparison should be between trained w1118 and the different trained transgenes (fig2) and then drug to no drug condition (fig 9), assuming these experiments were all performed together. Given there is no difference between the naïve male flies this comparison would be valid, and would take into better account the variability of the assay. Also, the number of flies assayed should be stated and, if less than 10, the individual scores should be plotted for the CI.

Also, when plotting the MI the authors appear to plot a single value, which I assume is the mean for the experiment, so that n=1. It would be better to score flies individually or repeat the experiment multiple times, so that error bars can be plotted.

-The lettering for Fig2 in the legend and the figure do not match.

-For Fig 7, can the authors show a control strain with no Aß as a reference for the ELISA.

-How many flies were scored for the eye phenotyping (Fig 1, 3 and 9)?

-What are the effects of acer mutation on the eye phenotypes induced by C99 or abtea42?  

 -Is it possible to measure the amount of Abeta in elav>C99 flies ? Because of the high amount of C99, it may be impossible to detect changes in C99 concentration (by western blot) in presence of captopril and losartan. However, it may be possible to detect changes in Abeta derived from C99 in these conditions. 
